# Ecosystem services relationship characteristics of the degraded alpine shrub meadow on the Qinghai‐Tibetan Plateau

**DOI:** 10.1002/ece3.10351

**Published:** 2023-07-23

**Authors:** Dawen Qian, Qian Li, Xiaowei Guo, Bo Fan, Yuting Lan, Mengke Si, Guangmin Cao

**Affiliations:** ^1^ School of Geography and Tourism Chongqing Normal University Chongqing China; ^2^ Northwest Institute of Plateau Biology Chinese Academy of Sciences Xining China

**Keywords:** alpine shrub meadow, ecosystem service, grassland degradation, Qinghai‐Tibetan Plateau, trade‐off

## Abstract

Alpine shrub meadows hold significant importance as grassland ecosystems on the Qinghai‐Tibetan Plateau (QTP). They provide a range of vital ecosystem services (ESs) and are commonly utilized as summer pastures by herders, resulting in short grazing periods and high grazing intensities. Unfortunately, these practices have led to varying degrees of degradation, thereby affecting the sustainable provision of ESs. However, the current knowledge regarding changes in ESs and their characteristics under the influence of degradation, particularly the differences between alpine shrub and alpine meadow ecosystems, is insufficient. To address this gap, this study aimed to investigate and analyse changes in four ESs within alpine shrub meadows across different levels of degradation, as well as explore their relationships. The research was conducted in a summer pasture located in the northeastern QTP. The findings revealed a substantial reduction of 85.9% in forage supply due to degradation in alpine shrub meadows. Moreover, regulating services experienced a decline followed by an increase in instances of heavy degradation. Trade‐offs were observed between provisioning and regulating services, while synergistic relationships were identified among different regulating services. Degradation exacerbated imbalances between provisioning and regulating services, whereas light degradation allowed for a better equilibrium between the two. Comparatively, alpine meadows exhibited higher levels of forage supply and carbon storage services, whereas alpine shrub ecosystems displayed greater nutrient supply and water retention services. It was observed that changes in ESs and relationship patterns within alpine shrub meadows were significantly influenced by the presence of alpine meadows. Consequently, safeguarding the structural integrity of alpine meadows and addressing conflicts over ESs is essential to ensure coordination and sustainability of ESs within alpine shrub meadows. The outcomes of this study provide valuable insights for ecosystem management and ecological restoration initiatives in alpine shrub meadows on the QTP.

## Graphical abstract

The imbalance between ecosystem services in alpine shrub meadow increases with the degradation of grassland. The evolution of the overall ecosystem services of alpine shrub meadow depends on changes in ecosystem services in the meadow layer. Conflicts between ecosystem services can be mitigated by maintaining the structural integrity of alpine shrub meadow.

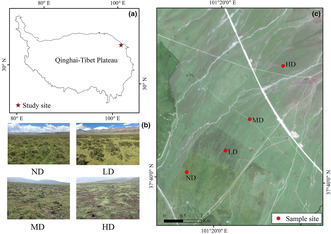

## 1 INTRODUCTION

Ecosystem service (ES) is defined as benefits arising from the interaction of multiple biotic or abiotic processes in an ecosystem that can be accessed directly or indirectly by humankind (Haines‐Young & Potschin, 2010; Millennium Ecosystem Assessment, 2005). For a long time, humans have worked to enhance the provisioning services that directly benefit them, such as food, timber and fibre, while ignoring the multifunctionality of ecosystems and the complex dynamic processes between them, thus leading to the unexpected or undesirable loss of other ESs (Bennett et al., 2009; Rodríguez et al., 2006; Zheng et al., 2019). The number of ES studies has grown significantly over the last decades, reminding humanity to focus on and protect ecosystems and to promote sustainable ecosystem development by improving management practices (Braat & de Groot, 2012; Cord et al., 2017; Lambers & Cong, 2022; Raudsepp‐Hearne et al., 2010). Understanding the relationships between ESs contributes to effective decision‐making on ecosystem‐based management (Zhang et al., 2020). In particular, identifying potential trade‐off conflicts will allow managers and other practitioners to minimize or eliminate losses to alternative services and formulate more effective, efficient and defensible decisions (Wong et al., 2015). Bennett et al. (2009) summarized three types of ES relationships: trade‐off, synergy and neutral. The term ‘trade‐off’ indicates that one service responds negatively to changes in the other service, ‘synergy’ indicates that both services have the same change direction, and a neutral relationship is defined when there is no interaction or no influence between the two services (Haase et al., 2012; Millennium Ecosystem Assessment, 2005).

The Qinghai‐Tibetan Plateau (QTP) is predominantly covered by alpine grasslands, making it the most dominant ecosystem type, encompassing 60% of the plateau's total area. These grasslands play a vital role in the ecological balance and functioning of the region (Dong & Sherman, 2015). It is the bearer of livestock activities and an essential material basis for the livelihoods of the 5 million herders on the plateau (Harris, 2010), as well as being able to store large amounts of carbon and nitrogen (Ni, 2002; Tian et al., 2006). Grassland degradation, which includes the decline of plant biomass, and the reduction in organic carbon and nutrient stocks, is defined as the succession process and results of the grassland ecosystem caused by interference of human and environmental factors (Liu et al., 2018). Over the past few decades, the degradation of alpine grasslands has emerged as a pressing ecological concern on the QTP. Extensive research has been dedicated to assessing the scale and severity of degradation, investigating the underlying mechanisms and understanding the repercussions on ESs.

At the regional scale, the assessment of alpine grassland degradation involves the utilization of remote sensing data and models. These tools enable the classification and estimation of the extent of grassland degradation based on vegetation indices or above‐ground biomass. The derived proportion of grassland degradation varies considerably, ranging from 30% to 90% (Li et al., 2018; Qin et al., 2019; Yang et al., 2016). At the local scale, the degree of alpine grassland degradation is determined by a variety of criteria including soil properties and plant community structural characteristics (Fayiah et al., 2020), but vegetation characteristics, including vegetation cover, biomass and the proportion of edible plants, are the most commonly used indicators (Miehe et al., 2019). The degradation of alpine grassland is generally considered to be a phenomenon caused by both climate change and human activities, specifically global climate change, overgrazing, unreasonable grazing management practices and excessive herbivory and soil disturbance from small mammals (Harris, 2010; Liu et al., 2018; Miehe et al., 2019).

The alpine shrub meadow is widespread on the shady mountain slopes of the QTP between 2500 and 4000 m and on the terraces of river valleys, occupying 4% of the total plateau area (Yashiro et al., 2010). The alpine shrub meadow, which encompasses both alpine shrub and alpine meadow vegetation types, serves as a crucial summer pasture for pastoralists on the QTP. Grazing activities predominantly occur between the months of June and September, when the vegetation is at its peak. This specific vegetation type thrives in foothills, shady slopes and depressions where favourable moisture conditions exist. It offers pastoralists substantial productive value by providing a reliable food source for domestic animals. In terms of ecological structure and function, the alpine shrub meadow exhibits distinct characteristics, including enhanced water retention, water supply, and carbon sequestration capabilities. These attributes differentiate it from the alpine meadow and contribute to its ecological significance on the plateau (Dai, Guo, et al., 2020; Li et al., 2016; Li, Li, Gao, et al., 2021). However, the short duration and the increasing intensity of grazing have led to the degradation of alpine shrub meadow, especially in the northeastern QTP (Dai, Guo, et al., 2020), and there is still insufficient attention to this phenomenon.

The degradation of grassland has caused a range of ecological problems, with a decline in plant cover and species richness, loss of soil nutrients and accelerated soil erosion (Dai, Yuan, et al., 2020; Li, Peng, et al., 2021; Liu et al., 2020), which in turn has led to a loss of ESs such as forage supply, carbon storage, nutrient cycling and water retention (Wang et al., 2015; Wen et al., 2013; Xu et al., 2019, 2021). Identifying the types of grassland ES relationships and understanding potential conflicts over grassland ESs, particularly under the impact of degradation, can be beneficial in developing strategies for grassland use and conservation. At present, research on grassland ES relationships can be divided into two main categories from the means of investigation. Firstly, it is based on remote sensing data and models to assess the characteristics of grassland ES relationships and their changes at global and regional scales through correlation analysis and spatial overlay analysis (Khosravi Mashizi et al., 2019; Pan et al., 2014; Petz et al., 2014). Secondly, based on field surveys, studies were carried out on the types of grassland ES relationships and their changes in the arid and semi‐arid zones, as well as QTP, and to assess the effects of grazing intensity, land management and restoration measures on grassland ES relationships (Fan et al., 2019; Li, Li, Liu, et al., 2021; Onatibia et al., 2015; Wu et al., 2017; Xu et al., 2019). Nevertheless, our current understanding of the effects of alpine shrub meadow degradation on ES remains limited, and the intricate relationships between these services remain poorly understood. Furthermore, it remains uncertain whether the responses of ES relationships to degradation differ between alpine shrub and alpine meadow ecosystems. Consequently, there is a crucial need for further research to elucidate the impact of alpine shrub meadow degradation on ES and explore potential variations in the responses of ES relationships between the two vegetation types. This knowledge gap hinders our ability to effectively manage and conserve these ecosystems and underscores the importance of scientific investigation in this field.

Therefore, two hypotheses are proposed for testing in this paper: (1) degradation of alpine shrub meadow alters their ESs and the relationships between ESs; (2) ES relationships in alpine meadow and alpine shrub show different characteristics during degradation. The aim of this paper is therefore to identify the changes of ESs and their relationships in alpine shrub meadow at different levels of degradation, and to compare the differences in ESs and their relationships between the alpine meadow and alpine shrub. The results of this paper will be useful for ES management and decision support in degraded alpine shrub meadow on the QTP.

## 2 METHOD AND DATA

### 2.1 Study site

**FIGURE 1:**
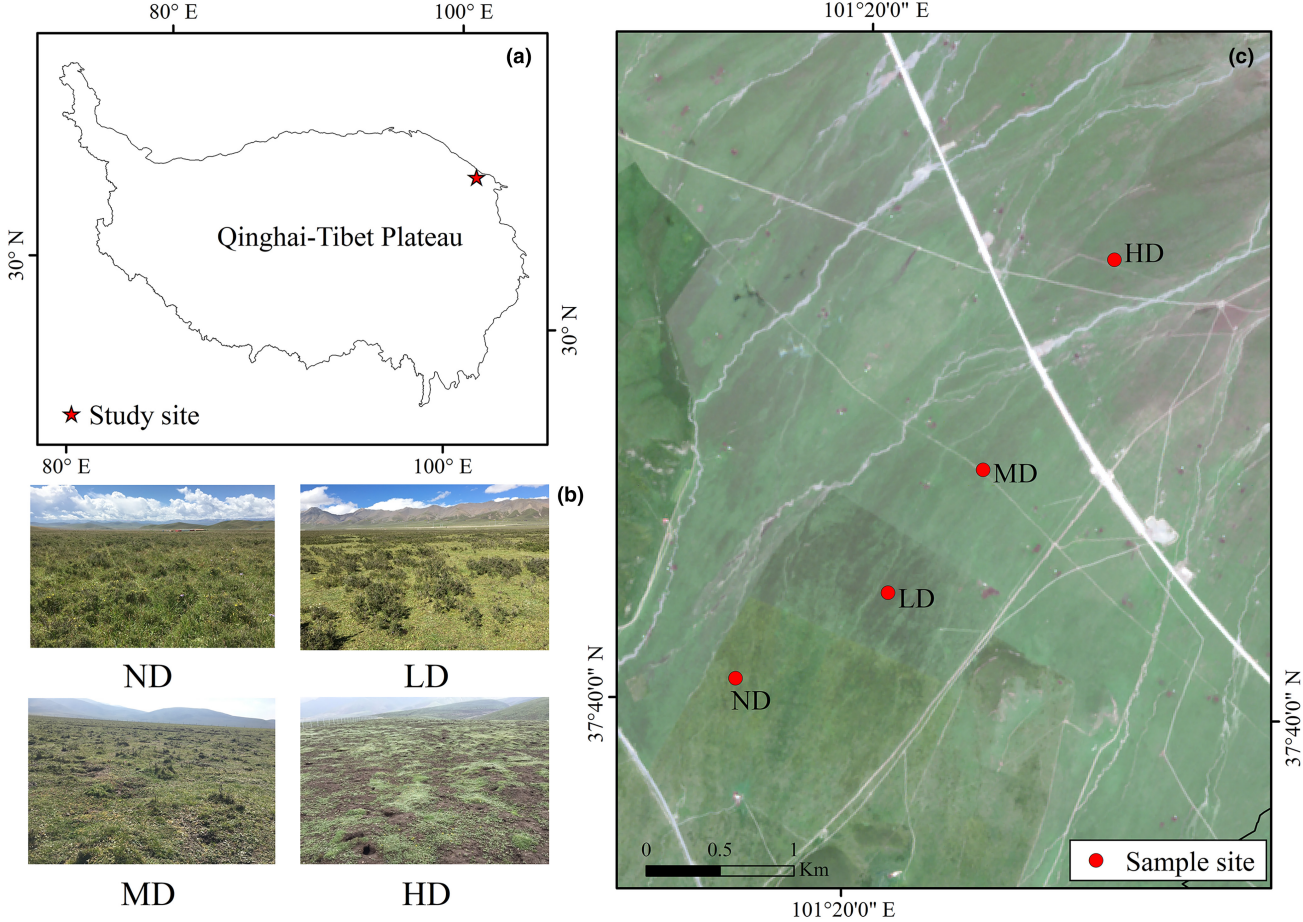
Study site location. (a) The location of the study site in the Qinghai‐Tibet Plateau; (b) Landscape photographs of different degraded sample sites. (c) Spatial distribution of different degraded sample sites. HD, heavy degradation; LD, light degradation; MD, moderate degradation; ND, non‐degradation.

The study area is located on the southern slopes of the Qilian Mountains (SSQM) in the northeastern part of the QTP, with an average altitude of 3320 m and flat terrain with an average slope of fewer than 5° (Figure 1). The average annual temperature and precipitation are −1.68°C and 590.1 mm, respectively, with 80% of the rainfall occurring mainly between May and September (Cao et al., 2007). The main vegetation type is alpine shrub meadow with a two‐layer structure. *Potentilla fruticosa* occupies the upper layer and *Korbresia humilis* meadow in the lower layer.

The alpine shrub meadow is used by local herders as summer pastures, grazing from June to September of the year. The main domestic animals are yaks (*Bos grunniens*) and Tibetan sheep (*Ovis aries*). Three different grazing regimes have existed in the area from north to south: communal grazing (no control of grazing intensity), joint family grazing (usually two or three families grazing together) and single‐family grazing. This grazing regime, which has been going on for at least 40 years according to the interviews with local herders. The long‐term practice of such different grazing management systems has led to different degrees of degradation of the alpine shrub meadow. The soil properties are relatively homogeneous, and the climatic conditions are similar, indicating that the observed degradation is primarily a consequence of different grazing management intensities (Dai, Guo, et al., 2020). We also noticed a high presence of plateau pika in heavily degraded grasslands, which might have indirectly contributed to the increased degradation of grasslands.

### 2.2 Field sampling

#### 2.2.1 Classification of alpine shrub meadow degradation levels

We classified the degradation of alpine shrub meadow into three levels based on *P. fruticosa* shrub cover and number of plant species: light degradation (LD), moderate degradation (MD) and heavy degradation (HD; Figure 1). In addition, we also selected a grazing exclusion site (100 m × 100 m) as non‐degraded (ND) alpine shrub meadow (Table 1; Dai, Guo, et al., 2020).

**TABLE 1:** Classification of alpine shrub meadow degradation levels.

Degradation level	Shrub coverage (%)	Species number
ND	50–60	24
LD	40–50	22
MD	5–10	26
HD	0	14

Abbreviations: HD, heavy degradation; LD, light degradation; MD, moderate degradation; ND, non‐degradation.

#### 2.2.2 Soil and plant sampling

In each degraded site, three plots (30 m × 30 m) were randomly distributed at a minimum interval of 20 m, with three replications of quadrat in each plot. In addition, nine quadrats were evenly distributed in an ‘S’ shape in the ND. Finally, the total number of quadrats was 36 and the samplings were carried out in early August 2020. Soil and plant surveys were carried out in alpine shrub and alpine meadows, where the quadrat sizes were 5 m × 5 m and 50 cm × 50 cm, respectively.

The above‐ground biomass of the alpine shrub was surveyed using the standard plant method: We randomly selected three large, medium and small shrubs in the quadrats based on the canopy size and subsequently cut the fresh branches and leaves as edible forage (Dai et al., 2019). The above‐ground biomass of the alpine meadow was determined using the standard harvesting method, which involves cutting the plants near the ground surface and separating the plant species into palatable and non‐palatable forage. The separated plant species were then weighed and recorded (Wu et al., 2017).

Using a 7‐cm diameter soil auger, soil samplings were carried out near the vegetation quadrats of alpine shrub and alpine meadow, respectively. Soil samples were obtained from 0 to 10 cm, 10 to 20 cm and 20 to 30 cm soil depth, for a total of 189 soil samples. A small portion (around 5–10 g) of the soil sample was first taken in an aluminium box and taken back to the laboratory to obtain soil water content using the drying method (Cui et al., 2019). The remaining soil samples were tested by the ferrous ammonium sulphate titrimetric method and the semi‐micro Kjeldahl method to get soil organic carbon and soil total nitrogen, respectively (Wu et al., 2017). Ultimately, we obtained soil moisture, soil organic carbon and soil total nitrogen content for alpine shrub and alpine meadows, respectively.

#### 2.2.3 Ecosystem services of alpine shrub meadow

Four key ESs were identified and assessed in this study, namely forage supply (FS), carbon storage (CS), water retention (WR) and nutrient supply (NS) services. Forage supply is of utmost importance as a provisioning service in alpine shrub meadows, serving as a primary resource for the sustenance of the surrounding pastoral communities. Carbon storage plays a critical role as a regulating service, representing the capacity of alpine shrub meadows to act as a ‘carbon sink’ in the face of climate warming. Water retention, on the other hand, assumes significant ecological importance in the context of alpine shrub meadows, serving as a vital regulating service within the broader scope of the SSQM. Lastly, nutrient supply represents the fundamental supporting services provided by alpine shrub meadows, serving as the underlying basis for the provision of other ESs.

Forage supply is represented by above‐ground palatable biomass (APB), while carbon storage, water retention and nutrient supply are represented by soil organic carbon (SOC), soil water content (SWC) and total soil nitrogen (TN), respectively (Khosravi Mashizi et al., 2019; Wu et al., 2017; Xu et al., 2019). To quantify the ESs, the corresponding indicators were calculated by averaging the values within the 0–30 cm soil depth. Specifically, forage supply service in alpine shrub meadows was determined by aggregating the forage supply services of both alpine shrub and alpine meadow ecosystems. On the other hand, the water retention, carbon storage and nutrient supply services of alpine shrub meadows were obtained by averaging the respective services of the two vegetation types.

### 2.3 ESs relationships

#### 2.3.1 Root mean of square error

The root mean of square error (RMSE) was applied to estimate the magnitude of trade‐offs between two or more ESs (Bradford & D'Amato, 2012).

(1) $$\text{RMSE}=\sqrt{\frac{1}{n-1}\sum_{i=1}^{n}{\left({\mathrm{ES}}_{i}-\bar{\mathrm{ES}}\right)}^{2}}$$

Where ES_*i*_ is the relative benefit of ecosystem service *i*, $\bar{\mathrm{ES}}$ is the average of all plots for ecosystem service *i*, *n* is the number of ecosystem services.

Relative ES was calculated as follows:

(2) $$\text{Relative}\;\mathrm{ES}=\frac{{\mathrm{ES}}_{i}-{\mathrm{ES}}_{i‐\min}}{{\mathrm{ES}}_{i‐\max}-{\mathrm{ES}}_{i‐\min}}$$

where ES_*i*‐max_ and ES_*i‐*min_ are the maximum and minimum values of the actual amount of the ecosystem service of type *i* in all sampling plots.

#### 2.3.2 Ecosystem trade‐off index

We used ecosystem trade‐off index (ETO) to quantify the degree of the trade‐offs among the different types of ES by using the following formula (Pan et al., 2013; Wu et al., 2017):

(3) $$\mathrm{ETO}=\ln\frac{\text{Relative}\;{\mathrm{ES}}_{\text{regulating}}}{\text{Relative}\;{\mathrm{ES}}_{\text{provisioning}}}$$

Relative ES_regulating_ and Relative ES_provisioning_ are the sum of the standardized values of regulating services and provisioning service, respectively. For −∞ < ETO < +∞, the positive value indicates that the regulating service has a dominant position in the trade‐off, while a negative value means that the provisioning service has a dominant position in the trade‐off. The larger the absolute value of ETO, the higher the degree of trade‐offs.

### 2.4 Statistical analysis

One‐way analysis of variance (ANOVA) was adopted to compare averages of ESs across the different degradation levels. A post hoc test of the Student–Newman–Keuls (SNK) test was performed to account for multiple comparisons at the *p* < .05 level. Spearman's rank correlation was used to study the relationship between ESs of alpine shrub meadow.

## 3 RESULTS

### 3.1 Changes in alpine shrub meadow ES at different degradation levels

**FIGURE 2:**
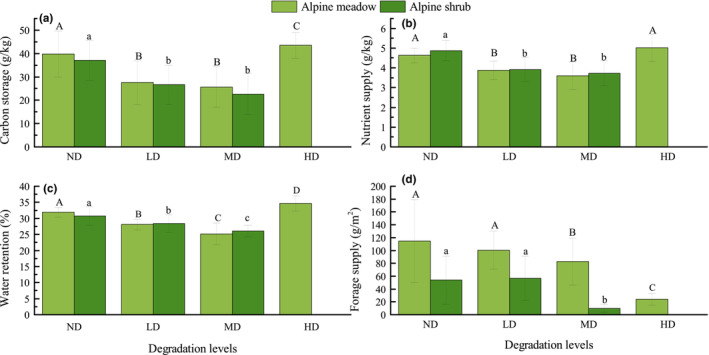
Ecosystem services changes of alpine meadow layer and alpine shrub layer at different degradation levels. (a) Carbon storage; (b) nutrient supply; (c) water retention; (d) forage supply. Different upper and lower case letters indicate significant differences in ES of alpine meadow and alpine shrub at different levels of degradation, respectively. Error bars represent 95% confidence interval. Alpine shrub disappeared from the surface at HD, no data are available for this level. HD, heavy degradation; LD, light degradation; MD, moderate degradation; ND, non‐degradation.

ESs were assessed at different levels of degradation in alpine shrub and alpine meadows, utilizing mean values within the 0–30 cm soil depth for carbon storage (CS), nutrient supply (NS) and water retention (WR) services. In both alpine shrub and alpine meadow ecosystems, CS exhibited a significant decrease (*p* < .05) at the LD level, with reductions of 12.2 and 10.5 g/kg, respectively. However, at the MD level, CS remained relatively stable. Notably, the CS of alpine meadow demonstrated a significant increase (*p* < .05) at the HD level (Figure 2a). Similar patterns were observed for NS, where both alpine shrub and alpine meadow experienced significant declines (*p* < .05) at the LD level, with reductions in 0.8 and 1 g/kg, respectively. At the MD level, NS did not show significant changes, while the NS of alpine meadow returned to undegraded levels at the HD level (Figure 2). The WR provided by alpine shrub and alpine meadows experienced continuous and significant decreases (*p* < .05) during the LD and MD levels, resulting in a cumulative reduction in 6.8% and 4.7%, respectively. However, the WR of alpine meadows has significantly increased (*p* < .05) during the HD, reaching its highest level (Figure 2). Both alpine shrub and alpine meadows experienced significant decreases (*p* < .05) in FS at the MD level, with reductions in 32.3 and 43.8 g/m^2^, respectively. Furthermore, the FS in alpine meadows exhibited a sustained and significant reduction (*p* < .05) during the HD level (Figure 2).

### 3.2 Relationships between ecosystem services of alpine shrub meadow

We investigated the relationships among various ESs within the alpine shrub meadow and found significant (*p* < .05) negative correlations between provisioning services and regulating services. Moreover, significant (*p* < .05) positive correlations were observed among the regulating services (Table 2).

**TABLE 2:** Relationships between different pairs of ecosystem services of alpine shrub meadow.

ES	CS	NS	WR	FS
CS	1	–	–	–
NS	0.877*	1	–	–
WR	0.814*	0.874*	1	–
FS	−0.382**	−0.251*	−0.296*	1

Abbreviations: CS, carbon storage; ES, ecosystem service; FS, forage supply; NS, nutrition supply; WR, water retention.

*Correlation is significant at the .05 level (two‐tailed). **Correlation is significant at the .01 level (two‐tailed).

**FIGURE 3:**
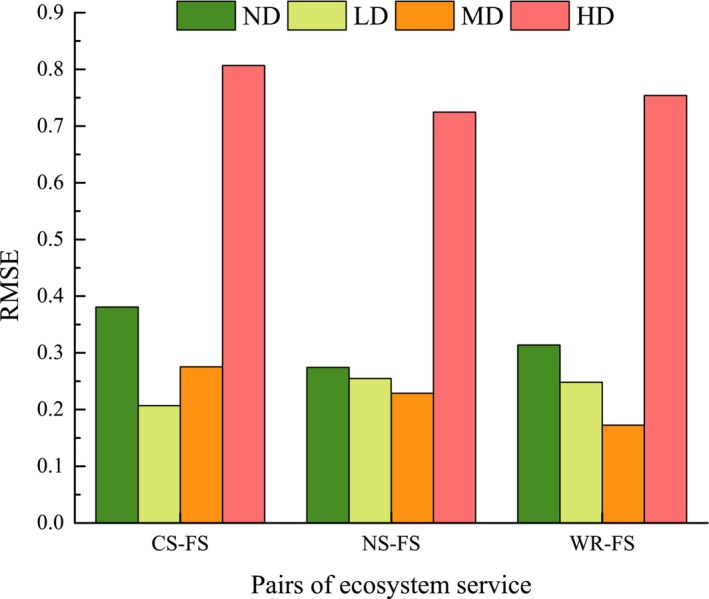
Root mean square error (RMSE) of alpine shrub meadow ecosystem services. CS, carbon storage; FS, forage supply; HD, heavy degradation; LD, light degradation; MD, moderate degradation; ND, non‐degradation; NS, nutrient supply; WR, water retention.

The RMSE between CS and FS services in alpine shrub meadow decreased in LD and increased continuously in MD and HD. The RMSE between NS, WR and FS services declined continuously in LD and MD and both increased in HD (Figure 3).

**FIGURE 4:**
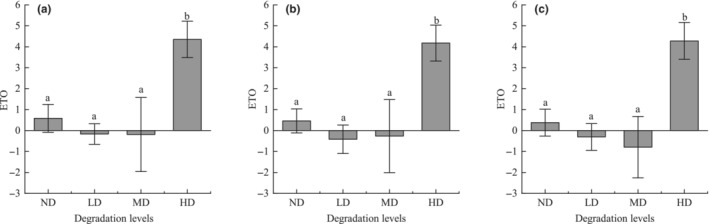
Degree of trade‐offs between provisioning and regulating services for (a) carbon storage/forage supply, (b) nutrient supply/forage supply and (c) water retention/forage supply. Different letters indicate significant differences in ES at different levels of degradation. Error bars represent 95% confidence intervals. HD, heavy degradation; LD, light degradation; MD, moderate degradation; ND, non‐degraded.

The trade‐off degree between regulating and provisioning services in alpine shrub meadow was greater than 0 in both ND and HD, indicating higher levels of regulating services compared to provisioning services. Conversely, in LD and MD levels, the trade‐off degree was consistently negative, indicating that provisioning services outweighed regulating services at these stages (Figure 4).

### 3.3 Differences in ESs between the alpine shrub and alpine meadow

There is a trade‐off relationship between the provisioning and regulating services of the alpine meadow, in which the trade‐off between FS and CS reached a significant level (*p* < .01), while the relationship between the provisioning and regulating services in the alpine shrub was unclear. The relationship between the regulating services of both the alpine meadow and the alpine shrub was synergistic, but the correlation coefficients of the alpine meadow were higher, and the synergistic relationship between the WR and NS reached a significant level (*p* < .05; Tables 3 and 4).

**TABLE 3:** Relationships between different pairs of ecosystem services of alpine meadow.

ES	CS	NS	WR	FS
CS	1.00	–	–	–
NS	0.72	1.00	–	–
WR	0.73	0.86*	1.00	–
FS	−0.33**	−0.15	−0.11	1.00

Abbreviations: CS, carbon storage; ES, ecosystem service; FS, forage supply; NS, nutrition supply; WR, water retention.

*Correlation is significant at the .05 level (two‐tailed). ** Correlation is significant at the .01 level (two‐tailed).

**TABLE 4:** Relationships between different pairs of ecosystem services of alpine shrub.

ES	CS	NS	WR	FS
CS	1	–	–	–
NS	0.77	1	–	–
WR	0.42	0.61	1	–
FS	−0.03	0.01*	0.20	1

Abbreviations: CS, carbon storage; ES, ecosystem service; FS, forage supply; NS, nutrition supply; WR, water retention.

*Correlation is significant at the .05 level (two‐tailed). **Correlation is significant at the .01 level (two‐tailed).

**FIGURE 5:**
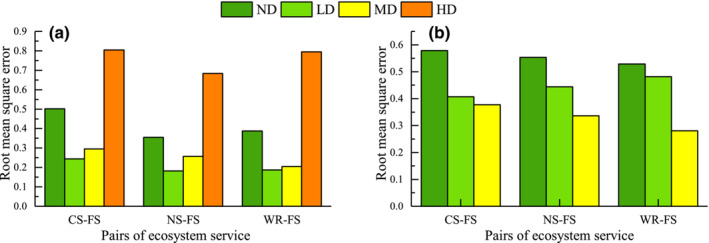
Root mean square error (RMSE) for ecosystem services in (a) alpine meadow and (b) alpine shrub. CS, carbon storage; FS, forage supply; HD, heavy degradation; LD, light degradation; MD, moderate degradation; ND, non‐degradation; NS, nutrition supply; WR, water retention.

The RMSE of the alpine meadow continued to increase in the MD and HD after decreasing in the LD, while the RMSE of the alpine shrub continued to decrease with degradation and was greater than the RMSE of the alpine meadow at the same degradation level (Figure 5).

**FIGURE 6:**
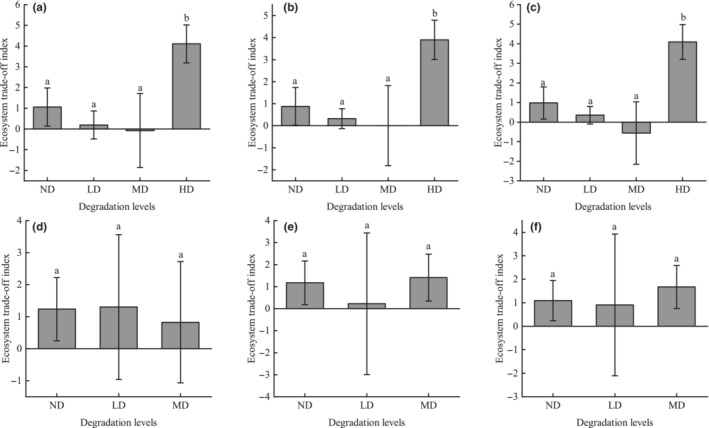
The degree of trade‐offs between provisioning and regulating services of meadow layer (a–c) and shrub layer (d–f). (a, d) Carbon storage/forage supply, (b, e) nutrient supply/forage supply, and (c, f) water retention/forage supply. Different letters indicate significant differences in ES at different levels of degradation. Error bars represent 95% confidence intervals. HD, heavy degradation; LD, light degradation; MD, moderate degradation; ND, non‐degraded.

The trade‐offs between regulating and provisioning services in the alpine meadow were greater than 0 in all levels except MD, and were significantly greater in the HD than in the other levels, where the trade‐offs between NS and FS were relatively small. The trade‐offs between regulating and provisioning services in the alpine shrub were greater than 0 at all levels, and the differences were not significant (Figure 6).

## 4 DISCUSSION

### 4.1 Effects of degradation on ESs in alpine shrub meadow

Grassland degradation can lead to the loss of ESs. In this study, the degradation of the alpine shrub meadow has adversely affected the delivery of multiple ESs. Specifically, provisioning services, such as forage supply FS, exhibited a decreasing trend with increasing degradation, reaching a minimum under heavy degradation. Additionally, regulating services, including CS, nutrient supply and water retention, also showed a decline in LD and MD. Previous studies have also confirmed the negative effects of grassland degradation on ESs such as the reduction in productivity, carbon storage, plant diversity and water retention services (Dai, Guo, et al., 2020; Wang et al., 2015; Wen et al., 2013). Notably, the provisioning services of grassland demonstrate the most pronounced response to degradation, with above‐ground biomass showing a decline as degradation intensifies (Li, Lyu, et al., 2021). Similarly, regulating services, such as water retention, nutrient cycling, carbon storage, also typically diminish with increasing degradation (Dai, Guo, et al., 2020; Dai, Yuan, et al., 2020; Wen et al., 2013).

The response of ESs to grassland degradation may exhibit a lag or even a rebound effect at certain stages. This phenomenon can be attributed to the multifaceted factors and intricate mechanisms influencing the formation and alteration of ESs. Additionally, interactions among different ESs further contribute to the complexity of their responses (Bennett et al., 2009; Lee & Lautenbach, 2016; Obiang Ndong et al., 2020). Therefore, solely assessing grassland degradation based on surface conditions does not necessarily correspond to an immediate decline in ESs. In fact, at specific stages, certain ESs may even increase instead. In the case of the alpine shrub meadow, the regulating services exhibited an upward trend during heavy degradation, with both carbon storage CS and water regulation WR significantly higher than in the non‐degraded ND level. These findings align with other studies that have reported that soil organic carbon and the soil water‐holding capacity remain at a high level in the heavy degradation (Guo et al., 2019; Wang et al., 2019; Zhang et al., 2018).

The well‐developed below‐ground root system of the alpine meadow, along with the slow accumulation and decomposition of roots, may account for the increase in regulating services observed in the HD level of the alpine shrub meadow. Soil organic carbon (SOC) is mainly derived from the decomposition and secretion of plant roots, the decomposition of shrub and meadow roots under heavy degradation may have increased the SOC content (Dai, Guo, et al., 2020; Gill & Jackson, 2000; Miehe et al., 2019; Rasse et al., 2005). Numerous studies have also established a positive correlation between SOC and soil moisture in grassland ecosystems (Dai, Guo, et al., 2020; Yang et al., 2014). These factors likely contribute to the enhanced delivery of ESs in the alpine shrub meadow under heavy degradation. Additionally, alterations in the physical properties of the soil can impact ESs. Notably, the significant presence of plateau pikas in the HD level has been observed, and their activities have led to reduced soil compaction and increased soil bulk, consequently enhancing soil porosity. This, in turn, improves water infiltration capacity and results in higher soil moisture levels (Dai, Guo, et al., 2020; Yang et al., 2014).

Both the alpine meadow and alpine shrub components play crucial roles in the alpine shrub meadow ecosystem by providing important provisioning and regulating services. The alpine meadow encompasses grasses, sedges and legumes, which are preferred forage options for livestock, thus serving as the primary source of forage supply service (Miehe et al., 2019; Wang et al., 2018). The well‐developed below‐ground root system of alpine meadow, along with root decay and decomposition, contributes to higher levels of organic carbon storage (Dai, Guo, et al., 2020; Li et al., 2011). On the contrary, the shrub component's root system exhibits a notable nitrogen fixation effect, comparable to the ‘fertile island effect’ observed in shrubs within arid and semi‐arid regions (Ding & Eldridge, 2021). This accounts for its elevated nutrient supply service during the LD and MD levels. Furthermore, previous studies have verified the enhanced water retention capacity of shrubs compared to meadows (Dai et al., 2021).

### 4.2 The relationships of ESs in alpine shrub meadow

The interactions among grassland provisioning and regulating services often give rise to conflicts and trade‐offs, while the relationships among different regulating services tend to be synergistic. In the context of our study on alpine shrub meadow, we identified a trade‐off between provisioning and regulating services, alongside a synergistic relationship among regulating services. Wu et al. (2017) documented trade‐offs between forage supply and soil nitrogen, as well as ecosystem carbon stock, in alpine grassland. Similarly, Fan et al. (2019) observed trade‐offs between herbage intake and biodiversity conservation, soil water content, nutrient cycling and soil carbon storage in Inner Mongolian grassland, while synergistic relationships existed between these regulating services. Therefore, similar to other grassland types, alpine shrub meadow also encounters imbalances in ESs that can impact its long‐term sustainability.

This study provides evidence that light degradation poses no immediate threat to the balance of ES relationships within the alpine shrub meadow. It demonstrates that light degradation can meet the demands for grassland production functions by pastoralists while still maintaining the functioning of regulating services. Previous studies have also supported the notion that moderate utilization of grassland does not cause detrimental effects to its ecosystems (Fayiah et al., 2020; Teng et al., 2020), and our findings further reinforced this perspective by examining ES relationships. However, with increasing degradation, the imbalance between provisioning and regulating services in the alpine shrub meadow becomes more pronounced, as observed in the study by Li, Lyu, et al. (2021).

The alpine meadow assumes a crucial role in shaping the ES relationships and trade‐offs within the alpine shrub meadow, serving as the focal point of ES conflicts. It exerts a greater influence on the overall ES relationships of the alpine shrub meadow, characterized by more pronounced correlations between its ESs and an escalating degree of trade‐offs with increasing degradation. The heightened demand for FS within the alpine meadow engenders imbalances in the relationships between its ESs. Thus, it becomes imperative to address the conflicts between forage supply and regulating services within the alpine meadow to ensure the sustainability of ESs in the alpine shrub meadow.

Preserving the structural integrity of both the alpine meadow and alpine shrub is paramount to safeguarding and enhancing the overall ESs provided by the alpine shrub meadow. In the design of management practices for the alpine shrub meadow, a central focus should be on effectively balancing or mitigating conflicts that may arise between the provisioning and regulating services of the alpine meadow. Furthermore, in the assessment of degradation and the undertaking of ecological restoration efforts within the alpine shrub meadow, careful consideration should be given to the time lag and resilience associated with changes in its regulating services. Leveraging the temporary increases in these services becomes an opportune strategy for restoring the multifunctionality of the grassland ecosystem.

## 5 CONCLUSION

This study aimed to investigate the changes in ESs and their relationships in a degraded alpine shrub meadow located on the northeastern QTP. The degradation of the alpine shrub meadows has resulted in a decline in ESs, particularly in the case of supporting services (FS). Surprisingly, regulating services have shown an increase under severe degradation conditions, potentially due to the slow accumulation and decomposition processes facilitated by the well‐developed root system of the meadow. These findings suggest a possible time lag in the response of alpine shrub meadow ESs to degradation. The analysis revealed trade‐offs and synergies that exist among provisioning and regulating services, as well as among different regulating services within the alpine shrub meadow ecosystem. Furthermore, the degree of trade‐offs has been found to increase with degradation, although light degradation can alleviate conflicts between provisioning and regulating services. While both alpine meadow and alpine shrub contribute significantly to the provision of ESs, the alpine meadow exhibits a greater influence on the relationships among these services. Therefore, it is crucial to preserve the structural integrity of the alpine shrub meadow to ensure the continued delivery of its ESs. In order to effectively manage and restore grasslands in the QTP, it is important to address the conflicts between provisioning and regulating services in the alpine meadow and comprehend the characteristics of variability in their regulating services. The results of this paper can provide insights and recommendations for the ecological restoration and management of ESs in the alpine shrub meadow ecosystem on the QTP.

## AUTHOR CONTRIBUTIONS

**Dawen Qian:** Conceptualization (lead); data curation (lead); funding acquisition (lead); investigation (equal); methodology (equal); software (lead); visualization (equal); writing – original draft (lead); writing – review and editing (lead). **Qian Li:** Conceptualization (supporting); formal analysis (equal); investigation (equal); methodology (supporting); resources (equal); supervision (equal); writing – review and editing (supporting). **Xiaowei Guo:** Investigation (supporting); resources (supporting); software (supporting); supervision (supporting); writing – review and editing (supporting). **Bo Fan:** Investigation (equal); resources (equal); validation (equal). **Yuting Lan:** Investigation (equal); validation (equal). **Mengke Si:** Investigation (equal); resources (supporting); validation (supporting). **Guangmin Cao:** Project administration (equal); resources (equal); supervision (equal); validation (supporting); writing – review and editing (supporting).

## CONFLICT OF INTEREST STATEMENT

No conflict of interest exists in the submission of this manuscript, and the manuscript is approved by all authors for publication.

## Data Availability

Data sets used in this study are available online in Dryad from DOI: 10.5061/dryad.p8cz8w9wf.
